# Increased chemoresistance *via* Snail–Raf kinase inhibitor protein signaling in colorectal cancer in response to a nicotine derivative

**DOI:** 10.18632/oncotarget.8049

**Published:** 2016-03-14

**Authors:** Tsai-Yu Lee, Chia-Lin Liu, Yun-Ching Chang, Shin Nieh, Yaoh-Shiang Lin, Shu-Wen Jao, Su-Feng Chen, Tsung-Yun Liu

**Affiliations:** ^1^ Institute of Environmental and Occupational Health Sciences, School of Medicine, National Yang-Ming University, Taipei, Taiwan, ROC; ^2^ Division of Colon and Rectum Surgery, Department of Surgery, Tri-Service General Hospital Songshan Branch, National Defense Medical Center, Taipei, Taiwan, ROC; ^3^ Division of Colon and Rectum Surgery, Department of Surgery, Tri-Service General Hospital, National Defense Medical Center, Taipei, Taiwan, ROC; ^4^ Graduate Institute of Life Sciences, National Defense Medical Center, Taipei, Taiwan, ROC; ^5^ Department and Graduate School of Pathology, National Defense Medical Center & Tri-Service General Hospital, Taipei, Taiwan, ROC; ^6^ Department of Otolaryngology-Head and Neck Surgery, Kaohsiung Veterans General Hospital, Kaohsiung, Taiwan, ROC; ^7^ Department of Dental Hygiene, China Medical University, Taichung, Taiwan, ROC

**Keywords:** nicotine, NNK, chemoresistance, Snail, colorectal cancer

## Abstract

A tobacco-specific component, 4-methylnitrosamino-1-3-pyridyl-1-butanone (NNK), is a major risk factor for many cancers. Recent reports have demonstrated that NNK exposure may be associated with tumor progression and chemoresistance in certain cancers. However, the underlying NNK-induced mechanism contributing to the aggressiveness of colorectal cancer (CRC) has not been thoroughly studied. In this study, we used HT29 cells treated with NNK to simulate the long-term exposure of cigarette smoke. A comparative analysis was performed to evaluate cell proliferation, migration, and invasion as well as epithelial-mesenchymal transition (EMT) markers and drug-resistance genes expression, cancer stem cell (CSC) properties, and anti-apoptotic activity. Signaling pathways related to chemoresistance were also investigated. As a result, NNK exposure dose-dependently stimulates cell proliferation, enhance abilities of migration and invasion, induce EMT phenomenon, and attenuate apoptosis. Furthermore, NNK exposure also promotes the capabilities of sphere formation, upregulation of Snail, and overexpression of CD133, Nanog, OCT4, and the drug-resistant genes. Knockdown of Snail results in upregulation of Raf kinase inhibitor protein (RKIP), increased apoptosis, reversal of EMT phenomenon, and reducation of expression of CSC markers, all of which contribute to a decrease of chemoresistance. Our study demonstrates a number of related mechanisms that mediate the effect of NNK exposure on increasing CRC therapeutic resistance via the Snail signaling pathway. Targeting Snail may provide a feasible strategy for the treatment of CRC.

## INTRODUCTION

Colorectal cancer (CRC) is a leading cause of cancer-related mortality worldwide [1]. Approximately 20% of newly diagnosed CRC cases present with local invasion and lymph node metastasis, and more than 50% of patients with early-stage CRC at initial diagnosis will develop metastatic foci [2]. Chemotherapy is used as an adjuvant therapy for resectable CRC or palliative therapy for advanced CRC to increase the survival time [3]. Despite substantial progress in both the diagnosis and the therapy in recent decades, the rate of local recurrence and distant metastasis remains as high as 15%–20%, and the 5-year overall survival rate remains lower than 10% [4]. Given the high rate of resistance resulting from a lack of complete response in a majority of CRC patients, novel molecular strategies for the enhancement of the conventional therapy for CRC are needed [5].

Tobacco use is a major public health issue worldwide [6]. The tobacco-related carcinogen nitrosamine, 4-methylnitrosamino-1-3-pyridyl-1-butanone (NNK), is a major component and the most potent carcinogen in cigarette smoke [7]. NNK is known to be associated with lung, oral, breast, gastric, pancreatic, and bladder cancers [8–11]. Among CRC patients, current smokers were found to have a 4-fold increased risk of developing hyperplastic and adenomatous polyps compared with people who had never smoked [12]. Ye et al. reported that the NNK stimulation of cell proliferation is dependent on 5-lipoxygenase and cyclooxygenase-2 expression in human colon cancer cell line [13]. Despite these studies, there is a lack of experimental evidence regarding the effect of tobacco smoking on CRC pathogenesis.

Cancer stem cells (CSCs) are also termed cancer-initiating cells due to their capacity for self-renewal, multilineage differentiation, and greater malignant potential [14]. The generation of CRC stem cells, involving the induction of epithelial-mesenchymal transition (EMT), has been postulated to play a critical role in CRC recurrence following chemotherapy [15, 16]. Therefore, EMT and CSC molecular pathways associated with chemoradiation resistance may provide insights into tumor survival mechanisms and identify novel targets for improved CRC treatment strategies [16]. NNK has been shown to activate multiple signaling pathways including activating kinase (Akt), mammalian target of rapamycin, extracellular signal-regulated kinase (ERK), and janus kinase [8]. NNK has been reported to enhance colon cancer metastasis through alpha-7 nicotinic acetylcholine receptor (α7-nAChR) and loss of epithelial cadherin (E-cadherin), a characteristic event in EMT, and its transcription repressors [17]. In addition, patients who continued cigarette smoking following surgery or chemotherapy have poorer prognosis and higher recurrent rates [18]. However, the mechanisms of the effect of NNK on promotion of CSC properties via EMT and the relationship between NNK and chemoresistance in CRC remain unclear.

Therefore, we aimed to determine the mechanisms of the effect of NKK on CRC pathogenesis including the contribution of NNK on EMT, CSC properties, anti-apoptosis and therapeutic resistance. Investigating the signaling pathways underlying the association between NNK exposure and CRC may provide novel treatments for the prevention of tumor progression in CRC.

## RESULTS

### Pro-proliferative and anti-apoptotic effect of NNK in CRC

**Figure 1:**
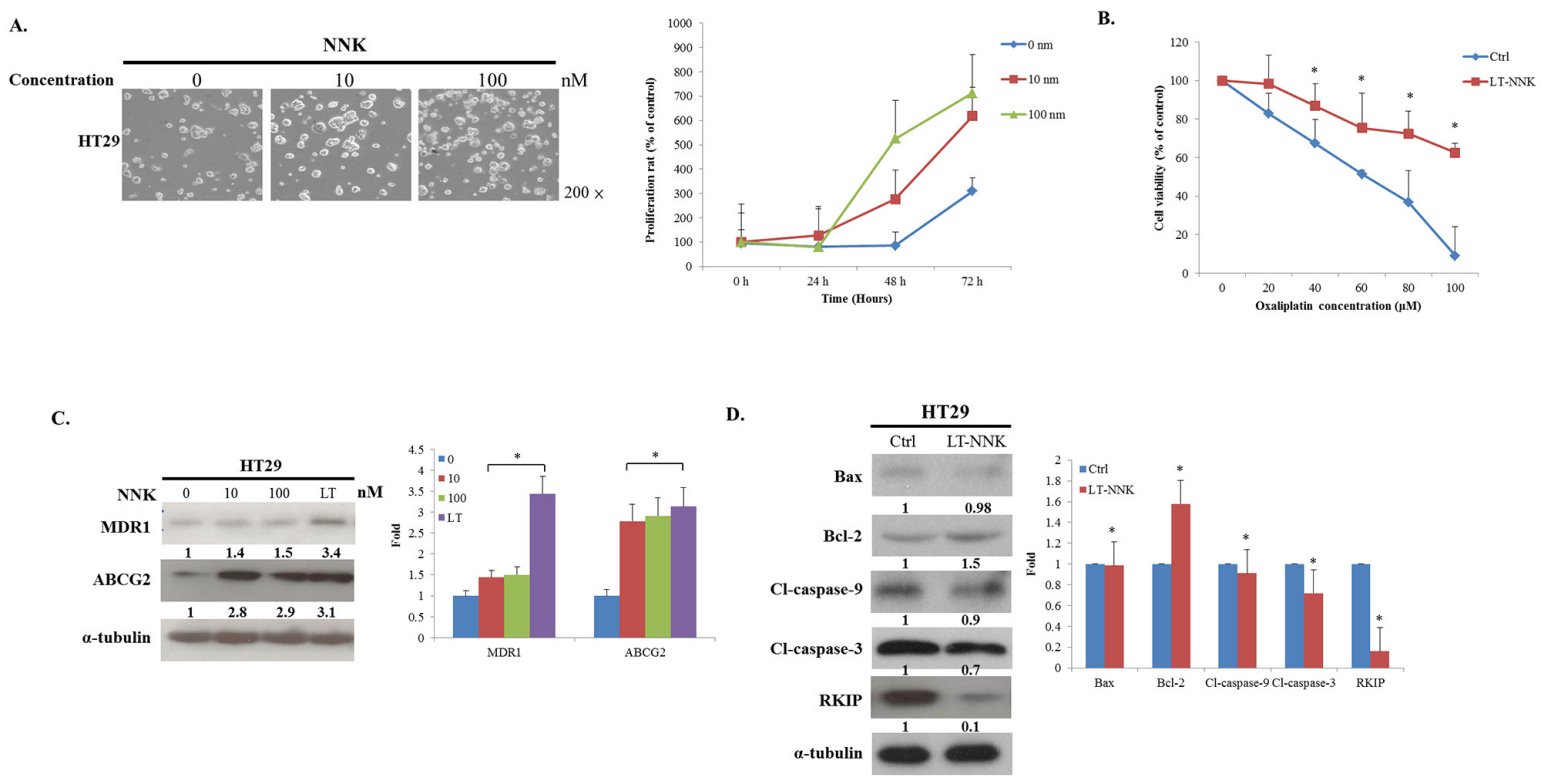
NNK exposure promoted cell proliferation, induced chemoresistance and increased anti-apoptosis **A.** NNK exposure stimulated the cell proliferation in both a dose- (0, 10, 100 nM) and time-dependent (24, 48, 72 h) manner (Magnification 200X) **B.** LT-NNK exposure induced chemoresistance with statistical significance in HT29 cells. **C.** LT-NNK exposure upregulated expression of MDR1 and ABCG2. **D.** Western blotting analysis demonstrated LT-NNK-treated cells conceded an anti-apoptotic property with downregulation of apoptotic-related proteins including Bax, caspase-9, caspase-3, along with decreased RKIP; and upregulation of Bcl-2 in HT29 cells.

To investigate the effect of NNK on the proliferation of human CRC cells, proliferation tests in HT29 cells were analyzed by 3-[4,5-dimethylthiazol-2-yl]-2,5-diphenyltetrazolium bromide (MTT) assay. Cells were seeded in 24-well plate and cultured with medium containing varying concentrations of NNK. As shown in Figure 1A, NNK significantly increased cell proliferation in both a dose- and time-dependent manner. In a further study, HT29 cells were treated with NNK for 3 weeks to simulate long-term exposure to cigarette smoke. Significant differences in chemosensitivity tests were found between long-term NNK exposure (LT-NNK)-treated cells and parent cells following exposure to varying doses of Oxaliplatin for 48 h (Figure 1B). LT-NNK exposure upregulated expression of multidrug resistance protein 1 (MDR1) and ATP-binding cassette sub-family G member 2 (ABCG2) (Figure 1C). Expression of B-cell lymphoma 2 (Bcl2)-associated X protein (Bax), cleavage caspase-9, cleavage caspase-3, and Raf kinase inhibitor protein (RKIP) was decreased in LT-NNK-treated cells compared with parent cells (Figure 1D). Furthermore, NNK was found to attenuate apoptosis. These data suggest NNK has pro-proliferative increased and antiapoptotic effects on CRC cells and may contribute, at least in part, to CRC chemoresistance.

### NNK enhanced migration and invasion via EMT

**Figure 2:**
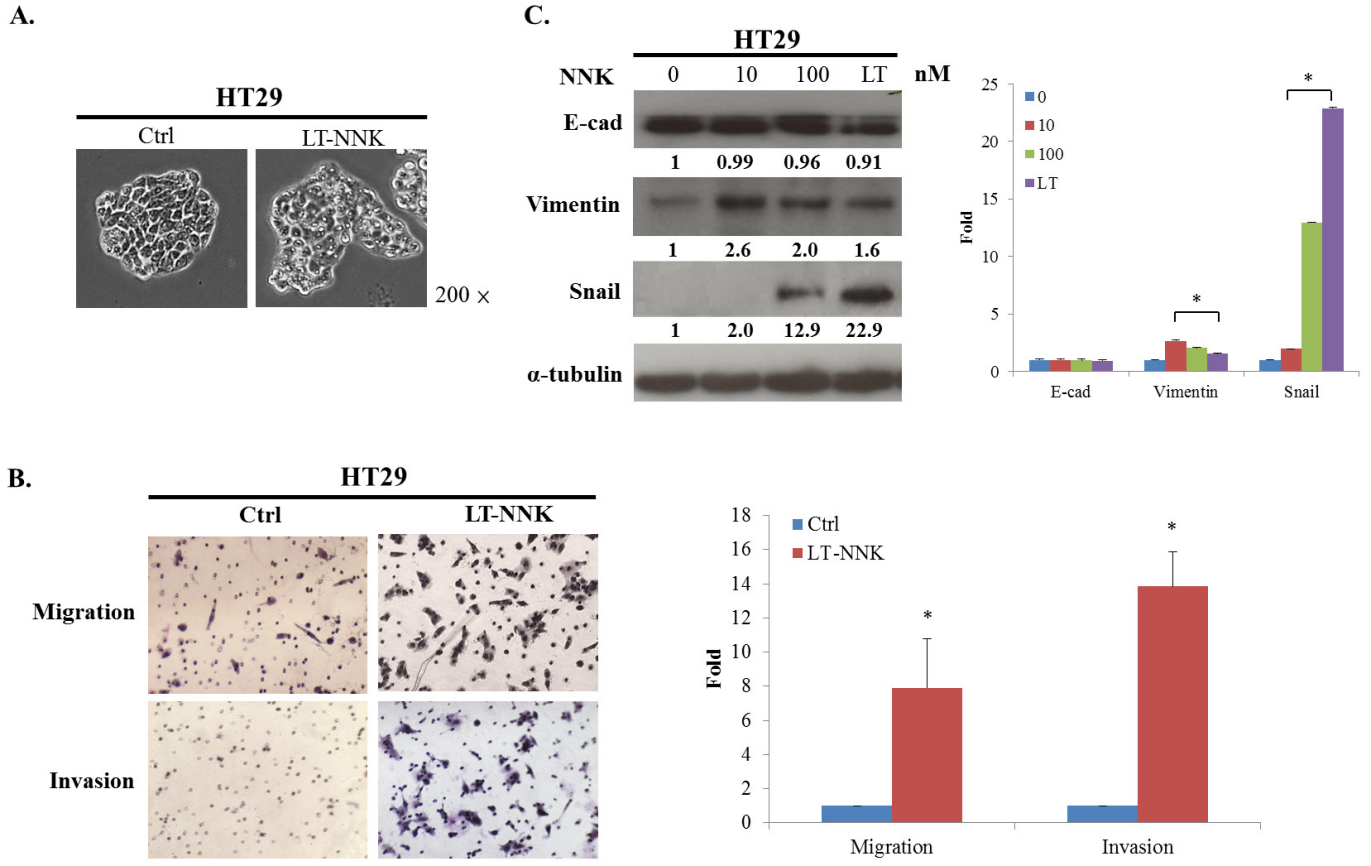
NNK exposure lead to EMT and enhanced the migration and invasion in HT29 cells **A.** LT-NNK exposure induced the EMT phenomenon with morphological transformation and alterations of cellular configuration in HT29 cells. **B.** LT-NNK exposure enhanced the abilities of migration and invasion. **C.** LT-NNK exposure altered EMT representative markers with decreased the expression of E-cadherin, increased the expression of vimentin, and significantly upregulated Snail signaling pathway in HT29 cells.

As EMT is a fundamental pathologic event in tumor progression [16], we further evaluated the expression of EMT in LT-NNK-treated cells. We first noticed that NNK exposure induced the EMT phenomenon, as shown by cell culture (Figure 2A). To evaluate the effect of NNK on CRC cell motility, migration and invasion assays were performed. LT-NNK exposure significantly increased migration and invasion properties of HT29 cells (*p* < 0.05) (Figure 2B). NNK exposure was found to induce EMT, as demonstrated by characteristic changes in cellular morphology and alterations in EMT marker expression including decreased expression of E-cadherin and increased the expression of vimentin and Snail (Figure 2C). Taken together, these data suggest that NNK stimulation induces characteristic cytological EMT changes in CRC cells leading to increased CRC cell migration and invasion.

### Enhanced CSC characteristics of LT-NNK-treated CRC cells

**Figure 3:**
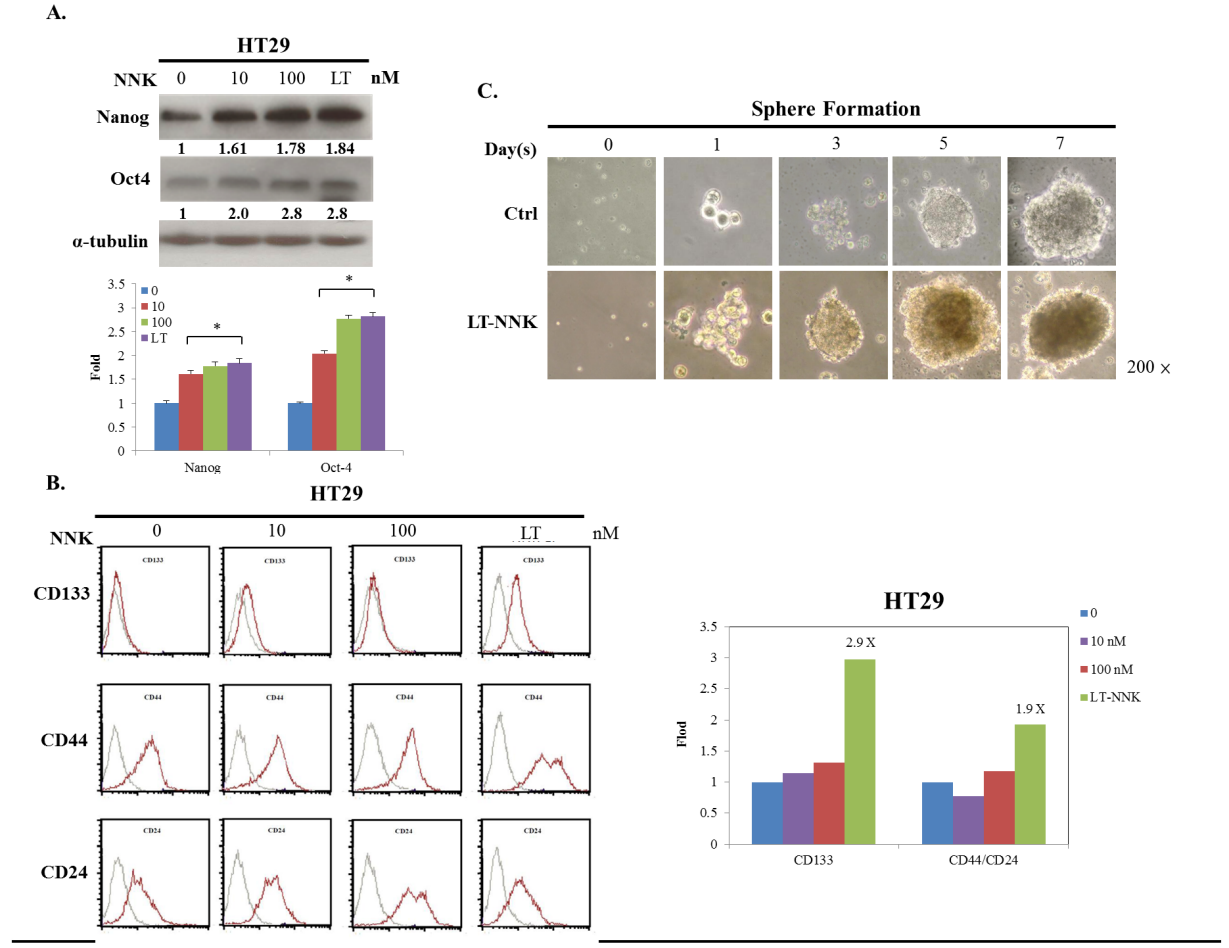
LT-NNK exposure enriched CSC properties with presentation of CSC-representative markers and sphere formation **A.** Overexpression of CSC-representative markers including Nanog and OCT4 was found in NNK-treated cells in a dose dependent manner, compared with control cells by Western blotting. **B.** LT-NNK exposure also demonstrates increased expression of CSC-representative markers including CD133, CD44 and CD24 by flow cytometry. **C.** Using a nonadhesive culture system, LT-NNK exposure is prone to form sphere, compared with the control cells.

The generation of stem cell-like cancer cells is associated with the activation of the EMT program [14, 16]. We further examined the effect of NNK on inducing CSC characteristics in CRC cells. Western blotting demonstrated upregulation of stem cell markers including Nanog and octamer-binding transcription factor 4 (OCT4) in LT-NNK-treated cells compared with parent cells (Figure 3A). Flow cytometric analysis of representative CSC markers demonstrated significant overexpression of cluster of differentiation 133 (CD133), cluster of differentiation 44 (CD44), and cluster of differentiation 24 (CD24) in LT-NNK-treated cells compared with parent cells (*p* < 0.05) (Figure 3B). HT29 cells demonstrated sphere-formation following LT-NNK exposure in a nonadhesive culture system with morphological transformations observed in spherical colonies. During the first 3–5 days of culture, cell clusters appeared as immature, floating spheroids that then transformed into well-formed spheres around day 7. By contrast, control cells produced irregular cell masses without a spheroid appearance (Figure 3C). These data indicate LT-NNK exposure induces CSC characteristics in CRC cells.

### Snail induced the promotion of EMT, anti-apoptosis, and CSC properties was induced by NNK in CRC cells

**Figure 4:**
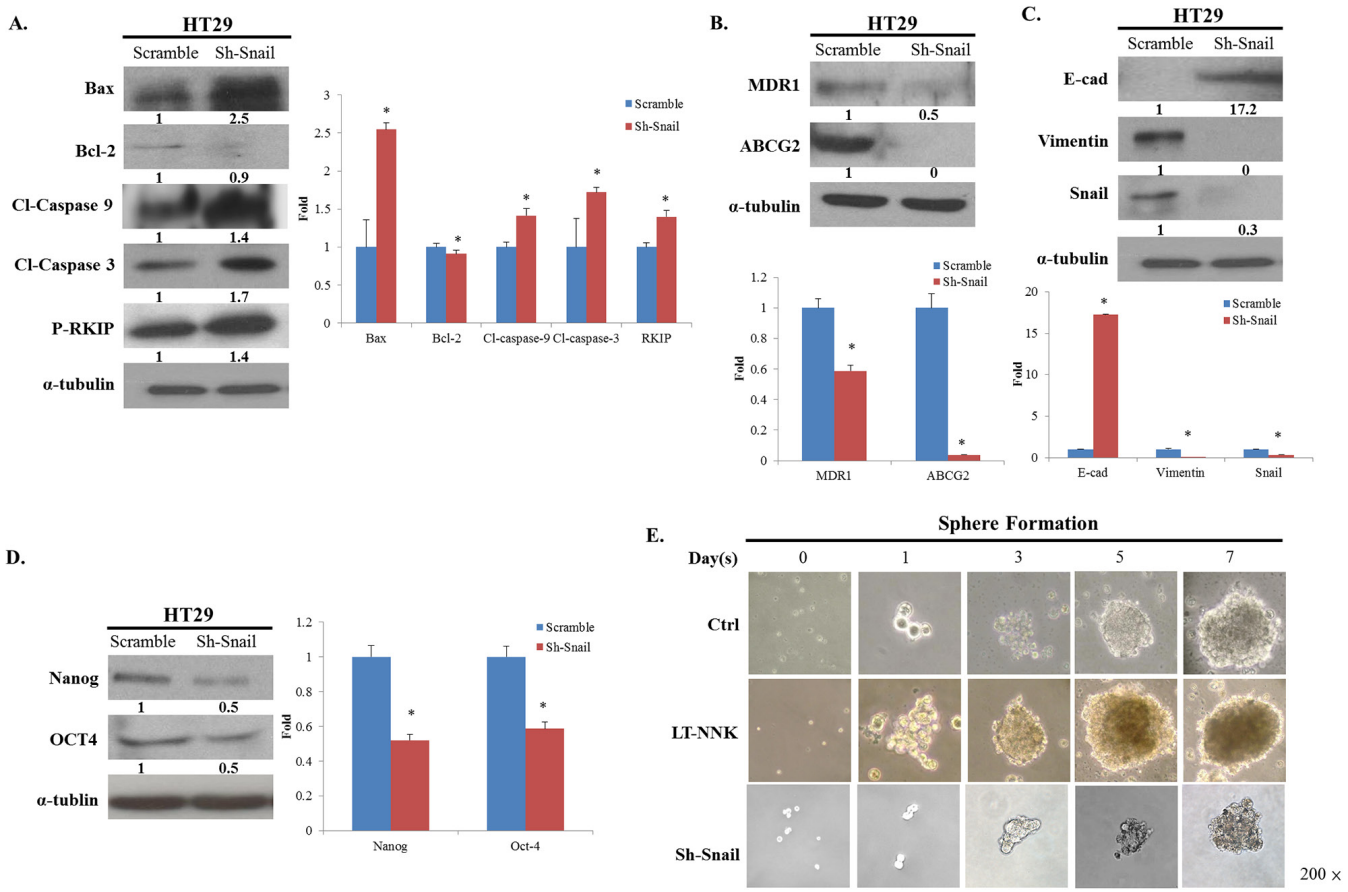
Knockdown of Snail restrained the expression of EMT, anti-apoptosis and CSC properties **A.** Knockdown of Snail altered the expression of apoptotic-related proteins and reverses the expression of RKIP. **B.** Knockdown of Snail decreased the expression of MDR1 and ABCG2. **C.** Knockdown of Snail increased the expression of E-cadherin, decreased the expression of vimentin, indicating a reversal of EMT phenomenon. **D.** Knockdown of Snail suppressed the expression of CSC properties with decreasing the expression of Nanog, OCT4. **E.** Knockdown of Snail declined the ability of sphere formation.

As the previous reports, the Snail signaling pathway has been implicated in NNK-induced EMT, reduced apoptosis and development of CSC characteristics [10, 19]. To determine the effects of NKK on the Snail signaling pathway in CRC cells, Snail knockdown was performed in LT-NNK-treated CRC cells. Snail knockdown led to altered expression of apoptosis-related proteins and attenuated expression of MDR1 and ABCG2 (Figure 4A and 4B). Increased expression of E-cadherin and decreased expression of vimentin were observed following treatment with sh-Snail, indicating reversal of EMT (Figure 4C). Inhibition of Snail in LT-NNK-treated CRC cells also suppressed sphere formation and expression of stem cell-related genes including Nanog and Oct4 (Figure 4D and 4E). These data indicate Snail contributes to induction of EMT, reduction in apoptosis, and promotion of CSC characteristics in CRC cells in response to NKK exposure.

## DISCUSSION

CRC is the third leading cause of cancer-related mortality in Taiwan [20]. Metastatic disease is the major cause of death in patients with CRC [21]. Long-term exposure to low doses of environmental carcinogens such as NNK contributes to increased risk of many cancers [9]. Epidemiologic studies have demonstrated that long-term cigarette smoking also increases CRC mortality [18]. However, the mechanisms of the effect of LT-NNK exposure and signaling pathways related to tumor progression of CRC were less discussed and required further investigation. In this study, we demonstrate the effect of long-term exposure to NNK at physiological levels inducing pathological changes and identified a potential therapeutic rationale by demonstrating Snail knockdown to noncytotoxic levels effectively suppresses tumor progression.

In this study, we found LT-NNK stimulated increased cell proliferation in both a dose- and time-dependent manner, increased cell migration and invasion, and decreased levels of apoptosis (Figure 1A, 1D and 2B). These findings and our previous report indicate that LT-NNK plays a critical role in tumor progression [22]. During the process of cancer invasion, detached malignant tumor cells overcome cell-cell adhesion and invade surrounding tissue involving a process termed EMT [19]. We found that LT-NNK exposure induces EMT and associated morphological changes and alters EMT marker expression, including downregulation of E-cadherin, overexpression of vimentin, and significantly increases the expression of Snail. Increasing evidence suggests EMT is fundamental mechanism underlying cancer aggressiveness and correlates with the development of CSC characteristics [23]. Our study also addressed the efficacy of preventive agents in preventing development of CSC characteristics. The generation of CSC, involving induction of the EMT program, has been postulated to have an important role in carcinogenesis and cancer recurrence following chemotherapy [24]. We demonstrated enrichment of CD133, CD44, and CD24-positive cell populations and increased sphere-forming capacity following cumulative exposure to NNK in colon cancer cells (Figure 3B and 3C). Overexpression of Nanog and OCT4 was observed in LT-NNK-treated cells compared with control cells (Figure 3A). Our study further examined the effect of LT-NNK exposure and demonstrated upregulation of two of the most significant chemoresistance genes, MDR1 and ABCG2, and increased Oxaliplatin-induced chemoresistance (Figure 1B and 1C), suggesting LT-NNK exposure also facilitates tumor progression. This is the first study to demonstrate the effects of LT-NNK exposure on the development of CSC characteristics and therapeutic resistance in CRC.

**Figure 5:**
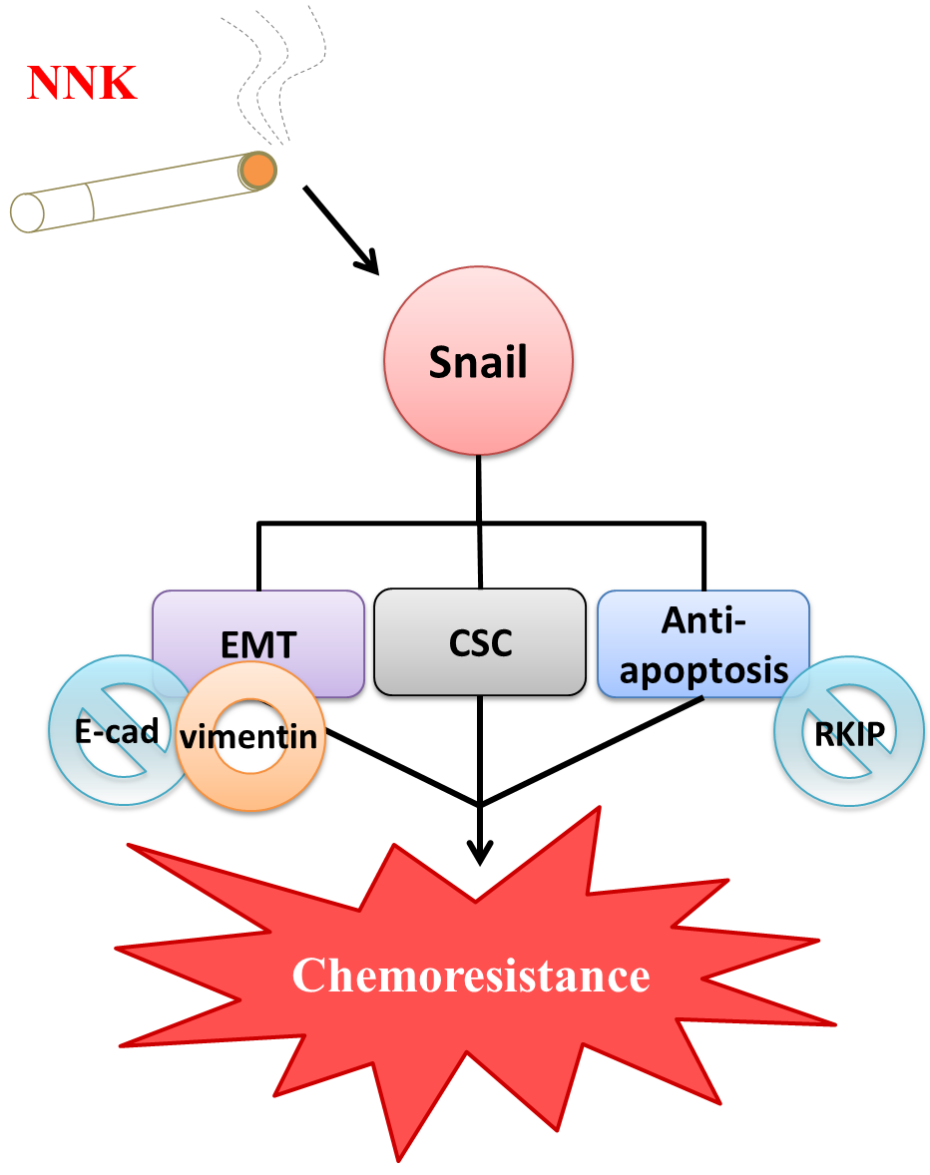
Diagrammatic illustration of a proposed model in terms of chemoresistance due to LT-NNK exposure in colon cancer cells LT-NNK exposure simulates the condition of cigarette smoking. LT-NNK exposure induced upregulation of Snail, with subsequent induction of EMT phenomenon characterized by alterated expressions of E-cadherin and vimentin, enrichment of CSC properties, and increase of anti-apoptosis via the Snail–RKIP signaling pathway, ultimately leading to chemoresistance and treatment failure.

Human colorectal carcinogenesis associated with chronic exposure to low doses of environmental carcinogens has been under-investigated. Our previous study demonstrated enrichment of CSCs associated with increased EMT characteristics in oral epithelial cells induced by nicotine is critical for oral squamous cell carcinoma (OSCC) tumorigenesis [22]. Downregulation of Snail attenuated tumorigenicity and the expression of stem cell markers in long-term nicotine-exposed OSCC cells. In this study, we found Snail was highly expressed in LT-NNK-exposed CRC cells (Figure 2C). The present study demonstrated inhibitory effects of Snail siRNA treatment on anti-apoptotic, EMT and CSC characteristics of CRC cells (Figure 4). The efficacy of Snail siRNA treatment in this LT-NNK exposure CRC model suggests that similar targeted approaches may have efficacy in CRC patients with a history of long-term smoking. This study demonstrated efficacy of Snail siRNA treatment in reducing the development of nicotine-stimulated CSC and EMT characteristics and blocking oncogenic progression in CRC cells. The effects of LT-NNK exposure or habitual heavy smoking on the development of chemoresistance in CRC, including alterations in Snail expression, induction of EMT, enrichment of CSC, and summarized in a diagram to show the major theme of our proposal by which LT-NNK exposure is responsible for the progression of the CRC (Figure 5).

In conclusion, application of the findings of this study may increase our understanding of the effect of chronic exposure to low doses of environmental and dietary carcinogens on colorectal carcinogenesis and lead to the identification of new approaches for reducing risk of CRC development. Molecular targeting of Snail may represent a new strategy for the treatment of CRC in patients with a history of long-term smoking. Avoidance or cessation of cigarette smoking increases the efficacy of chemotherapy in CRC conferring improved prognosis.

## MATERIALS AND METHODS

The methods were performed following the protocols of our laboratory as reported previously [22, 25–31].

### Testing reagents and drugs

The 4-(Methylnitrosamino)-1-(3-pyridyl)-1-butanone (NNK) was purchased from the ChemSyn Laboratories (Lenexa, KS, USA ) and the Oxalip^®^ (Oxaliplatin) was provided by the TTY Biopharm (Taipei, Taiwan, ROC)

### Preparation of cell line and cell viability

The cell line HT29 cells (HTB-38^™^, American Type Culture Collection, ATCC^®^, Rockville, MD, USA) were isolated from human colon adenocarcinoma. The cells were maintained in Roswell Park Memorial Institute (RPMI) 1640 medium supplemented with 10% fetal bovine serum (FBS), penicillin (100 U/mL), and streptomycin (100 μg/mL) in a humidified incubator at 37°C and 5% CO_2_ [22, 29]. The cell viability was determined by MTT assay (Sigma-Aldrich, St. Louis, MO, USA) [22]. The NNK was added at a dose rate of 0, 10, or 100 nM for 0, 24, 48, or 72 h.

### Induction of long-term NNK (LT-NNK) cells

The HT29 parent cells were plated at a density of 5×10^4^ live cells/10-cm dish and were treated with NNK at the concentration of 50 nM for three weeks. The medium was changed every other day [22].

### Migration and invasion assay

The migration ability was determined with a micropore chamber assay (6.5 mm Transwell^®^ with 8.0 μm Pore Polyester Membrane Insert, #3464, Corning Incorporated, NY, USA) and the invasion ability was determined with a modified Boyden chamber [22, 30]. For the invasion assay, the Matrigel–medium (1:2) mixture was supplied onto the membrane of the upper chamber before the cells were seeded. Then the cells were suspended in RPMI 1640 medium and plated in the upper chamber. The culture medium containing 0.5% serum was added in the lower chamber. The cells were harvested in a humidified incubator at 37°C for 12 h for the migration assay or 24 h for the invasion assay. The cells on the upper side of the filter were removed with cotton swabs carefully. The migratory or invaded cells on the lower surface of the filter were fixed in 4% formaldehyde and stained with hematoxylin. The number of cells was counted using a light microscope. Data from three independent experiments performed in triplicate are expressed as mean ± SD.

### Western blot analysis

Western blot was performed following the standard protocol [22, 31]. The cell lysates were separated by sodium dodecyl sulfate polyacrylamide gel electrophoresis (SDS-PAGE) and were transferred to the polyvinylidene fluoride membranes. After the membranes were blocked, the diluted primary antibodies, MDR1 (MAB4163, 1:1000, Chemicon, Temecula, CA, USA ), ABCG2 (MAB4155, 1:1000, Millipore, Billerica, MA, USA), α-Tubulin (sc-5286, 1:1000, Santa Cruz Biotechnology, Santa Cruz, CA, USA), E-cadherin (sc-7870, 1:1000, Santa Cruz Biotechnology, Santa Cruz, CA, USA), Snail (sc-28199, 1:1000, Santa Cruz Biotechnology, Santa Cruz, CA, USA), Nanog (sc-81961, 1:1000, Santa Cruz Biotechnology, Santa Cruz, CA, USA), Oct4 (GTX101497, 1:1000, Gene Tex, San Antonio, TX, USA), in Tris-buffered saline with Tween (TBST) buffer containing 3% non-fat milk were added to the membranes and incubated at 4°C overnight. On the second day, the goat anti-mouse conjugated secondary antibody was added at room temperature for 1 h. The immunoblots were developed using an enhanced chemiluminescence system and visualized on X-ray film.

### Flow cytometry

The cells were suspended in 1 ml PBS by trypsin and stained with primary antibody, CD133 (#3663, 1:200, Cell Signaling, Danvers, MA, USA), CD44 (MS-668, 1:200, Thermo, Waltham, MA, USA) and CD24 (sc-7034, 1:200, Santa Cruz Biotechnology, Santa Cruz, CA, USA), at room temperature for 1 h. After labeling and washed with PBS, the cells were stained with FITC- or PE-labeled secondary antibody in the dark for 30 min and analyzed using a BD FACSCalibur™ flow cytometer (BD Biosciences, San Diego, CA, USA) [22, 25].

### Sphere culture

The sphere culture was performed using a non-adhesive culture system [22, 25]. The cells were plated in 10-cm plastic wares at a density of 5×10^4^ live cells/10-cm dish with non-adhesive surfaces by coating with agarose thin films. The culture medium was changed every other day till the spheres formation.

### Chemosensitivity assay

A total of 1×10^6^ cells were seeded in a 10-cm dish and were treated with 0, 20, 40, 60, 80, and 100 μM Oxaliplatin (Oxalip^®^, TTY Biopharm, Tapiei, Taiwan, ROC) for 48 h. The relative survival fraction of cells was determined by MTS assay using the CellTiter 96^®^ AQueous One Solution Cell Proliferation Assay kit (Promega, Madison, WI, USA) [22].

### Lentivirus production and transfection of target cells

The Snail expression of HT29 cells was knocked down using the short hairpin RNA (shRNA) lentiviral particles and shRNA-expressing U6 lentivirus system (Santa Cruz Biotechnology, Santa Cruz, CA, USA). Transfection of target cells was performed as described previously [22, 32]. HT29 cells were selected with 2 μg/ml puromycin.

### Statistical analysis

The independent Student's t test or ANOVA was used to compare the continuous variables between groups, whereas the χ^2^ test was applied for the comparison of dichotomous variable. The level of statistical significance was set at 0.05 for all tests. The statistical analysis was done with the software PASW Statistics v18 (Armonk, NY, USA).
